# Repurposing INCI-registered compounds as skin prebiotics for probiotic *Staphylococcus epidermidis* against UV-B

**DOI:** 10.1038/s41598-020-78132-5

**Published:** 2020-12-09

**Authors:** Arun Balasubramaniam, Prakoso Adi, Do Thi Tra My, Sunita Keshari, Raman Sankar, Chien-Lung Chen, Chun-Ming Huang

**Affiliations:** 1 Department of Biomedical Sciences and Engineering, National Central University, Taoyuan, Taiwan; 2 Department of Life Sciences, National Central University, Taoyuan, Taiwan; 3 Institute of Physics, Academia Sinica, Nankang, Taipei, Taiwan; 4 Division of Nephrology, Landseed International Hospital, Taoyuan, Taiwan

**Keywords:** Biotechnology, Cancer, Microbiology, Molecular biology, Health care

## Abstract

Repurposing existing compounds for new indications may facilitate the discovery of skin prebiotics which have not been well defined. Four compounds that have been registered by the International Nomenclature of Cosmetic Ingredients (INCI) were included to study their abilities to induce the fermentation of *Staphylococcus* *epidermidis* (*S. epidermidis*), a bacterial species abundant in the human skin. Liquid coco-caprylate/caprate (LCC), originally used as an emollient, effectively initiated the fermentation of *S. epidermidis* ATCC 12228, produced short-chain fatty acids (SCFAs), and provoked robust electricity. Application of LCC plus electrogenic *S. epidermidis* ATCC 12228 on mouse skin significantly reduced ultraviolet B (UV-B)-induced injuries which were evaluated by the formation of 4-hydroxynonenal (4-HNE), cyclobutane pyrimidine dimers (CPD), and skin lesions. A *S. epidermidis* S2 isolate with low expressions of genes encoding pyruvate dehydrogenase (*pdh*), and phosphate acetyltransferase (*pta*) was found to be poorly electrogenic. The protective action of electrogenic *S. epidermidis* against UV-B-induced skin injuries was considerably suppressed when mouse skin was applied with LCC in combination with a poorly electrogenic *S. epidermidis* S2 isolate. Exploring new indication of LCC for promoting *S. epidermidis* against UV-B provided an example of repurposing INCI-registered compounds as skin prebiotics.

## Introduction

Drug repurposing or repositioning is an effective approach to rapidly identify novel indications from known compounds^1,2^. There have been numerous successful cases of repurposed drugs, including sildenafil citrate (Viagra) as a medicine for erectile dysfunction and pulmonary arterial hypertension and raloxifene hydrochloride (Evista) as a treatment for osteoporosis in postmenopausal women^3^. Repurposing drugs, including Remdesivir^4^ and Dexamethasone^5^, to discover potential forms of treatment for severe acute respiratory syndrome coronavirus 2 (SARS-CoV-2) is actively ongoing. Skin diseases affect half of the world's population and novel drugs for treatments of skin diseases are in high demand. The skin is the largest organ in humans and continuously protects the humans from the harmful environment^6,7^. Over 100 distinct species, which contribute to making up a total of 1 million microbes in the skin microbiome, conquer every square centimeter of human skin^6,8^. Our previous studies have demonstrated that fermentation of skin probiotic bacteria generated beneficial metabolites, such as short-chain fatty acids (SCFAs), that can attenuate skin disorders^9^. For example, *Staphylococcus epidermidis* (*S. epidermidis*), a common member in the human skin microbiome, can fermentatively metabolize carbon-rich molecules as prebiotics to yield SCFAs against pathogenic *Staphylococcus aureus* (*S. aureus*)^10^.

Prebiotics were originally defined by Gibson and Roberfroid in 1995 as nondigestible food ingredients^11^. This definition was later revised in^12^ and^13^ as “Glucose-based dietary fibers and non-carbohydrate substances including polyunsaturated fatty acid (PUFA) have been used as prebiotics for gut bacteria”. Prebiotics for bacteria in the skin and other human organs are not yet defined. Prebiotics can provide probiotic bacteria as carbon sources to initiate the fermentation and produce SCFAs as acetate and butyrate^14^. It has been reported that oxidation of acetate or butyrate served as an electron donor to discharge electron to electron acceptors^15^. The gene-encoding proteins in the extracellular electron transfer (EET) family of homologs are believed to be present in both Gram-negative and Gram-positive bacteria^16^. Unlike Gram-negative bacteria, Gram-positive bacteria, not having an outer membrane, carry a cell envelope with a textured peptidoglycan layer and teichoic acids that are thought to be poorly electrogenic^17^. However, a flavin-based EET process has been recently identified in Gram-positive bacteria to produce electricity^16^ by activation of type II NADH hydrogenase, which can catalyze electron exchange from cytosolic NADH to a quinone derivative such as quinone demethylmenaquinone (DMK)^16^. Our recent studies demonstrated that *S. epidermidis* can mediate glycerol as a source to generate electricity and enhanced bacterial resistance to UV-B^18^.

In this study, we selected four carbon-rich molecules that have been listed on the International Nomenclature of Cosmetic Ingredients (INCI) to examine their prebiotic activities. The liquid coco-caprylate/caprate (LCC) with C8-10 fatty acid connected to C12-C18 fatty alcohols is currently used as an emollient on ultraviolet (UV) filter absorbance^19^. Isononyl isononanoate (ININ) (C_18_H_36_O_2_) is an ingredient in cosmetics and personal care products as an emollient, texture enhancer, and plasticizer^20^. Polyethylene glycol (PEG)-150 distearate (PDS) [(C_2_H_4_O)_n_.C_36_H_70_O_3_] is a thickening agent for shampoo products^21^. PEG-150 pentaerythrityl tetrastearate (PETIS) (C_77_H_148_O_8_) has been used for increasing viscosity of an aqueous agent in cosmetics^22^. To screen these four carbon-rich molecules for repurposing, we examined their prebiotic activities for induction of fermentation of *S. epidermidis*. It has been documented that *S. epidermidis* can mediate fermentation to down-regulate UV-B-induced inflammation in mouse skin^9^. We thus further explored the mechanism by which repurposing carbon-rich molecules as skin prebiotics influence the skin damage induced by UV-B. UV radiation which provokes free radical formation, making it a primary risk factor for skin cancer^23^. It has been reported that UV, in particular UV-B, radiation can up-regulate the local neuroendocrine axes, induce the release of hormones to circulation, activate the central hypothalamic–pituitary–adrenal axis, and reset body hemostasis against skin disorders including cancers, aging and autoimmune diseases^24^. Repurposing may facilitate the discovery of new mechanisms of action for INCI-registered ingredients as skin prebiotics against UV injuries.

## Methods

### Ethics statement

All animal protocols and experiments have been approved by the National Central University (NCU), Taiwan. Experiments were conducted in accordance with the protocols (NCU-106-016, 19 December 2017) of the Institutional Animal Care and Use Committee (IACUC) of National Central University (NCU). Female ICR mice (8–9 weeks old) were purchased from the National Laboratory Animal Center Taipei, Taiwan. CO_2_ sedation was used to sacrifice mice in an encased chamber. All human study protocols were approved by Institutional Review Board (IRB) (No. 19-013-B1, 22 May 2019) and Ethics Committee of Landseed International Hospital, Taiwan. The methods followed for skin swab sampling procedure were carried out in accordance with relevant guidelines and regulations of IRB which was approved by Landseed International Hospital, Taiwan. Skin swabs were collected from three healthy subjects and informed consent was obtained from all study participants.

### Bacterial fermentation

*Staphylococcus epidermidis* ATCC 12228 in tryptic soy broth (TSB) (Sigma, St. Louis, MO, USA) was cultured overnight at 37 °C. Bacterial growth was determined at 600 nm wavelength (OD_600_). The bacterial pellet was collected after centrifugation at 5,000 × g for 10 min, resuspended with 1 × PBS and diluted to 10^7^ CFU/ml before further incubation in rich media [1.5 g/l KH_2_PO_4_, 10 g/l yeast extract (Biokar Diagnostics, Beauvais, France), 2.5 g/l K_2_HPO_4_, 3 g/l TSB, and 0.002% (w/v) phenol red (Merck, Darmstadt, Germany)] at 37 °C. For fermentation, bacteria in rich media in the presence of 2% LCC, ININ, PDS or PETIS (TNJC corporation, Chiayi, Taiwan) were incubated for 12 h. The color change of phenol red from red to yellow indicated bacterial fermentation, which was quantified by measurement of OD_562_. Bacteria alone or rich media with or without LCC, ININ, PDS or PETIS served as controls. To examine the effect of LCC on the bacterial growth, *S. epidermidis* ATCC 12228 [10^7^ colony-forming unit (CFU)/ml)] was incubated with 2% LCC or phosphate buffer saline (PBS) for 12 h at 37 °C. After incubation, bacteria were serially diluted 1: 10^0^–1:10^5^ in a 96 well plate. 10 μl of serially diluted bacteria were dropped on a TSB agar plate for CFU counts.

### Electricity detection

Electricity produced by *S. epidermidis* was detected in vitro using a chamber equipped with cathode and anode. A carbon felt (2.5 cm × 10 cm) and a carbon cloth (10 cm × 10 cm) (Homy Tech, Taoyuan, Taiwan) were utilized to fabricate anode and cathode., respectively. The cathode was wrapped up to a Nafion membrane N117 (6 cm × 6 cm) (Homy Tech), which served as a proton exchange membrane (PEM). Copper wires were used to connect anode and cathode with external resistance (1,000 Ω)^18^. *S. epidermidis* in the presence or absence of LCC, ININ, PDS or PETIS was pipetted on the surface of the anode. Electricity was recorded by the changes in voltage (mV) against time (min) using a digital multimeter (Lutron, DM-9962SD, Sydney, Australia). The recorded voltages in every 10 s were used for plotting a graph.

### Cyclic voltammetry

The three-electrode autolab potentiostat (PGSTAT 128 N, Metrohm Autolab, Utrecht, Netherland) was used for conducting cyclic voltammetry. The screen-printed carbon electrode (SPCE) (SE-100, Zensor R&D, Taichung, Taiwan) served as a working electrode with a working area of 5 mm. The Ag/AgCl electrode and platinum electrode acted as the reference against applied potential and counter electrode, respectively. All electrodes were purchased from Metrohm Autolab. *S. epidermidis* (10^7^ CFU/μl) in the presence or absence of 2% of LCC, ININ, PDS or PETIS was drop-coated on the surface of a working electrode. The potential windows were inspected between − 0.8 and 0.2 V at 0.005 V/s. PBS at 7.4 pH was used as an electrolyte. The potentiostat was operated using Autolab Nova 2.0 software (https://metrohm-autolab.com/Products/Echem/Software/Nova.html/; Metrohm Autolab, Utrecht, Netherland).

### Extraction of bacterial RNA

RNA was extracted from overnight cultured *S. epidermidis* ATCC 12228 or a S2 isolate (10^7^ CFU/ml). The cultured bacteria were centrifuged at 5,000 × g for 10 min and the pellet was collected. RNA in bacterial pellet was extracted using a total RNA mini purification kit (Biokit, Miaoli, Taiwan) and quantified by UV spectrophotometry in a Synergy HT multi-mode microplate reader (BioTek Instruments Inc., Highland Park, Winooski, Vermont, USA).

### Real-time qPCR (RT-qPCR)

RT q-PCR was used to analyze the expression of genes encoding pyruvate dehydrogenase (*pdh*), phosphate acetyltransferase (*pta*) and intracellular adhesion A (*ica A*) in *S. epidermidis* ATCC 12228 and S2 isolate. RNA (1 ng) was converted to cDNA using an iScript cDNA Synthesis Kit (Bio-Rad, Hercules, CA, USA). The cDNA was served as a template in StepOnePlus RT PCR System (Thermo Fisher Scientific, Waltham, MA, USA), which was executed using Power SYBR Green and PCR Master Mix (Thermo Fisher Scientific). The primer-Blast tool (https://blast.ncbi.nlm.nih.gov/Blast.cgi/; Rockville Pike, Bethesda MD, USA) from the National Center for Biotechnology Information (NCBI) was used for designing all primers. Total one step RT-PCR reaction condition was fixed for 40 cycles as follows: 95 °C for 10 min followed by 95 °C for 15 s, 60 °C for 60 s, and 72 °C for 30 s. Gene expression was normalized with the 16S rRNA gene. The cycle threshold (2^−ΔΔCt^) was implemented to analyze the relative expression of genes. The designed primers for all genes were shown in Table S2.

### Gas chromatography-mass spectrometry (GC–MS) analysis

*Staphylococcus epidermidis* ATCC 12228 (10^7^ CFU/ml) with 2% LCC in rich media was cultured for 24 h and then centrifuged at 5,000 × g for 10 min. Supernatants of bacterial culture were collected and filtered through 0.22 µm filters. The GC–MS protocol for SCFAs detection were obtained following the method^25^.

### UV-B exposure

Mouse hair in the dorsal skin was removed using Nair cream (Church and Dwight, Ewing Township, NJ, USA) one day before experiments started. The dorsal skin of each mouse was exposed to 100 mJ/cm^2^ UV-B irradiation using a UV lamp (Model EB-280C, Spectronics Corp., Westbury, NY, USA) twice a week, followed by subsequent application of 10^7^ CFU/ml *S. epidermidis* with or without 2% LCC three times per week for two weeks. The images of mouse skin were captured on day 0, 7 and 14. Skin lysates and sections from dorsal skin (1 cm^2^) was prepared. Skin sections were stained with hematoxylin and eosin (H&E) and visualized using the Olympus BX63 microscope (Olympus, Tokyo, Japan).

### Western blotting

Tissue Protein Extraction Reagent (T-PER) (Thermo Fisher Scientific) was used for preparing the skin lysates. Protein concentrations of skin lysates were measured by a bicinchoninic acid (BCA) assay (Bio-Rad). Skin lysates (30 μg) were loaded to a 10% sodium dodecyl sulphate–polyacrylamide gel electrophoresis (SDS-PAGE) gel and transferred to a PVDF membrane (Millipore Sigma, Burlington, MA, USA). The membrane was blocked with 5% (w/v) non-fat milk, and incubated with primary antibodies to cyclobutane pyrimidine dimer (CPD) (1:1,000; Cosmo Bio, Tokyo, Japan), 4-hydroxynonenal (4-HNE) (1:2,000; Abcam, Cambridge, MA, USA), or β-actin (1:5,000; ACE Biolabs, Taoyuan, Taiwan) overnight at 4 °C. The membrane was subsequently incubated with secondary antibodies goat anti-rabbit or anti-mouse IgG (H + L) horseradish peroxidase (HRP) (1:5000; ACE Biolabs) for 1 h. Protein bands in membranes were developed using chemiluminescent detection reagent (Thermo Fisher Scientific) and visualized by an Omega Lum C Imaging System (Gel Co., San Francisco, CA, USA). ImageJ software (https://imagej.nih.gov/ij/index.html/; National Institutes of Health, Bethesda, MD, USA) was employed to quantify the intensities of protein bands.

### Statistical analysis

GraphPad Prism 8 (https://www.graphpad.com/; GraphPad Software, La Jolla, CA, USA) software was employed for data analysis by unpaired t-test. The significant difference was considered by *P-*values observation as follows: *P-*values of < 0.05 (*), < 0.01 (**), and < 0.001 (***). The mean ± standard deviation (SD) was obtained from at least three separate experiments.

## Results

### INCI-registered compounds function as skin probiotics for induction of SCFA production and bacterial electricity

**Figure 1:**
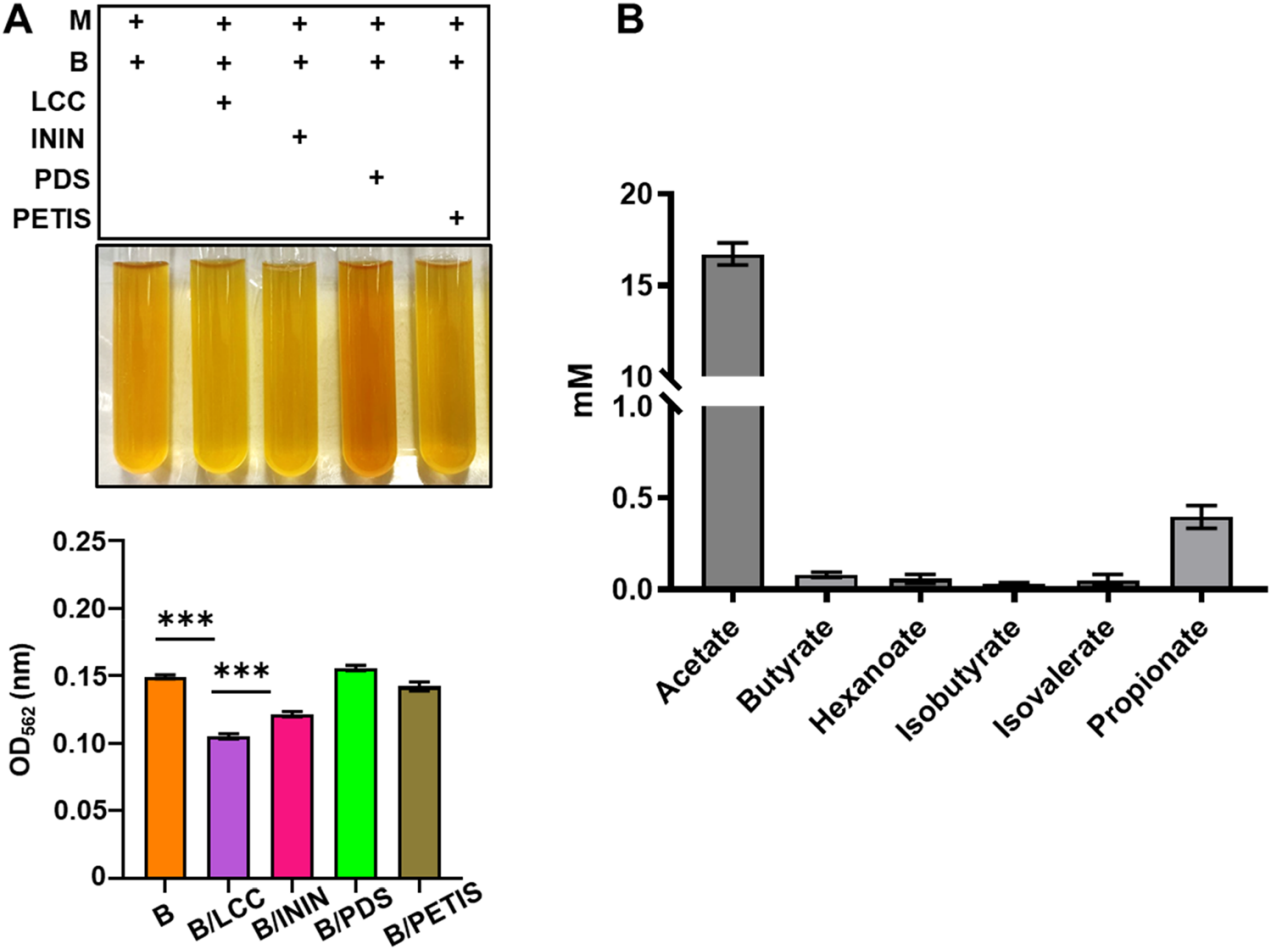
LCC fermentation of *S. epidermidis.* (**A**) *S. epidermidis* ATCC 12228 (10^7^ CFU/ml; **B**) was incubated for 12 h in rich media in the presence or absence of 2% LCC, ININ, PDS or PETIS. The prevalence of fermentation was indicated by colour change of phenol red from red to yellow and quantified by OD_562_. (**B**) *S. epidermidis* ATCC 12228 (10^7^ CFU/ml) in the presence of 2% LCC in rich media was cultured for 24 h. The levels (mM) of six SCFAs (acetate, butyrate, hexanoate, isobutyrate, isovalerate and propionate) in fermentation media were quantified. Data are the mean ± SD from three separate experiments. ***P < 0.001 (two-tailed t-test).

**Figure 2:**
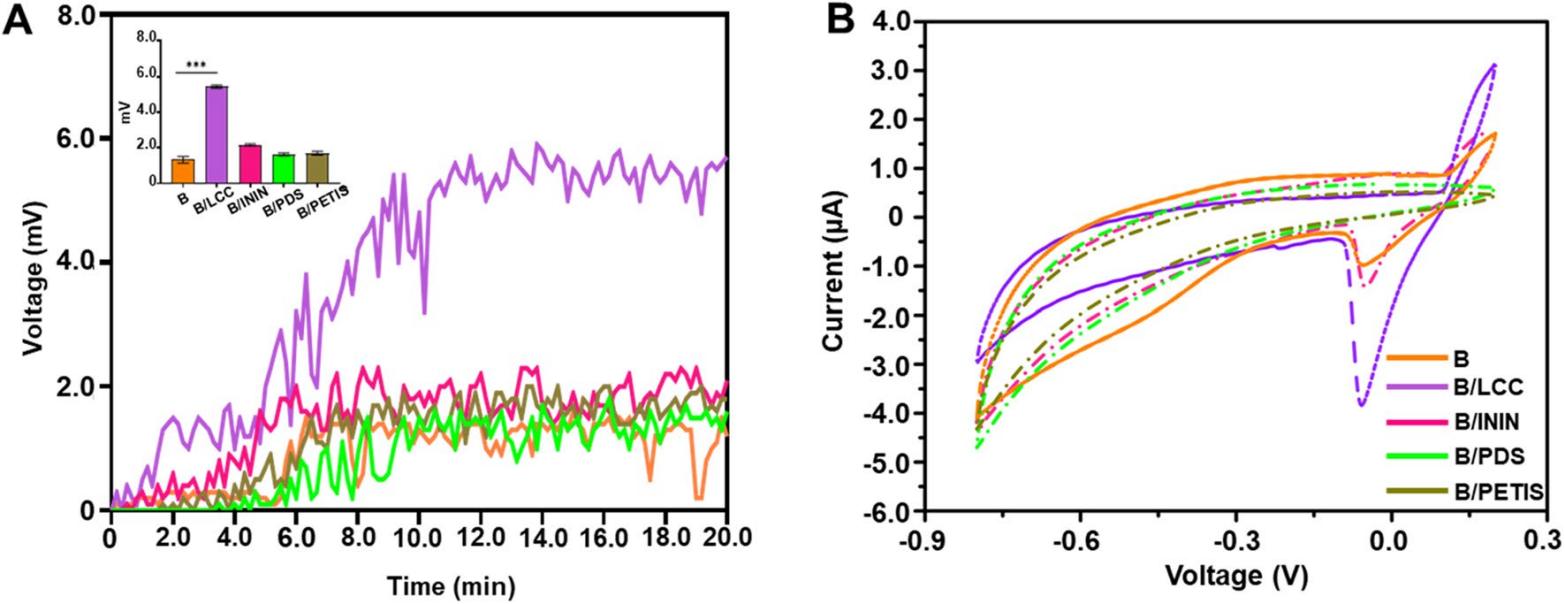
Electricity production by LCC fermentation of *S. epidermidis.* (**A**) The electricity measured by voltage changes (mV) was recorded for 20 min after pipetting *S. epidermidis* alone or with LCC (B/LCC), ININ (B/ININ), PDS (B/PDS) or PETIS (B/PETIS) on the surface of an anode. (**B**) Cyclic voltammetry was employed to measure the current (µA) generated by various experimental conditions above. Data presented the mean ± SD from three separate experiments. ***P < 0.001 (two-tailed t-test).

Our previous studies have demonstrated the fermentation and electrogenic activities of *S. epidermidis* in the presence of glycerol as a carbon source^18^. To examine if INCI-registered compounds can act as skin prebiotics that provides carbon sources to induce fermentation of skin bacteria, we cultured *S. epidermidis* ATCC 12228 (10^7^ CFU/ml), a non-biofilm forming skin bacterium, in rich media containing phenol red with 2% each individual INCI-registered compound including LCC, ININ, PDS, and PETIS for 12 h. Media with bacteria alone served as controls. A change in color of phenol red from red to yellow and a significant reduction of the optical density of 562 nm (OD_562_) due to low pH values^26^ in the culture media of the *S. epidermidis* in the presence of 2% each individual INCI-registered compound served as indications of bacterial fermentation. As shown in Fig. 1A, 2% LCC and ININ, compared to PDS and PETIS, induced significant fermentation of *S. epidermidis* by detection of yellowish media and OD_562_ reduction. The reduction of OD_562_ induced by LCC was greater than that by ININ. To validate the occurrence of fermentation, six SCFAs including acetate, butyrate, hexanoate, isobutyrate, isovalerate, and propionate in the media of LCC fermentation were detected by GC–MS analysis (Fig. 1B). A high amount of acetate (> 15 mM) was produced by LCC fermentation of *S. epidermidis*. SCFAs, especially acetate and butyrate, have been proved as potent electron donors during the EET process in bacteria^27–29^. We thus investigated the electrogenic activity of *S. epidermidis* in the presence of INCI-registered compounds. Changes in voltages in an in vitro chamber and current values detected by cyclic voltammetry^30^ were used to monitor bacterial electricity. Compared to media with *S. epidermidis* alone, media with *S. epidermidis* plus ININ, PDS or PETIS did not elicit high voltage changes and currents. By contrast, a robust increase in voltage changes with a peak voltage of approximately 6 mV and currents was detected in the media with *S. epidermidis* plus LCC (Fig. 2). The result provided evidence for repurposing INCI-registered LCC as a prebiotic to provoke the fermentation and electricity production of skin *S. epidermidis* bacteria.

### Topical application of *S. epidermidis* plus LCC reduced UV-B-induced the formation of 4-HNE and CPD

**Figure 3:**
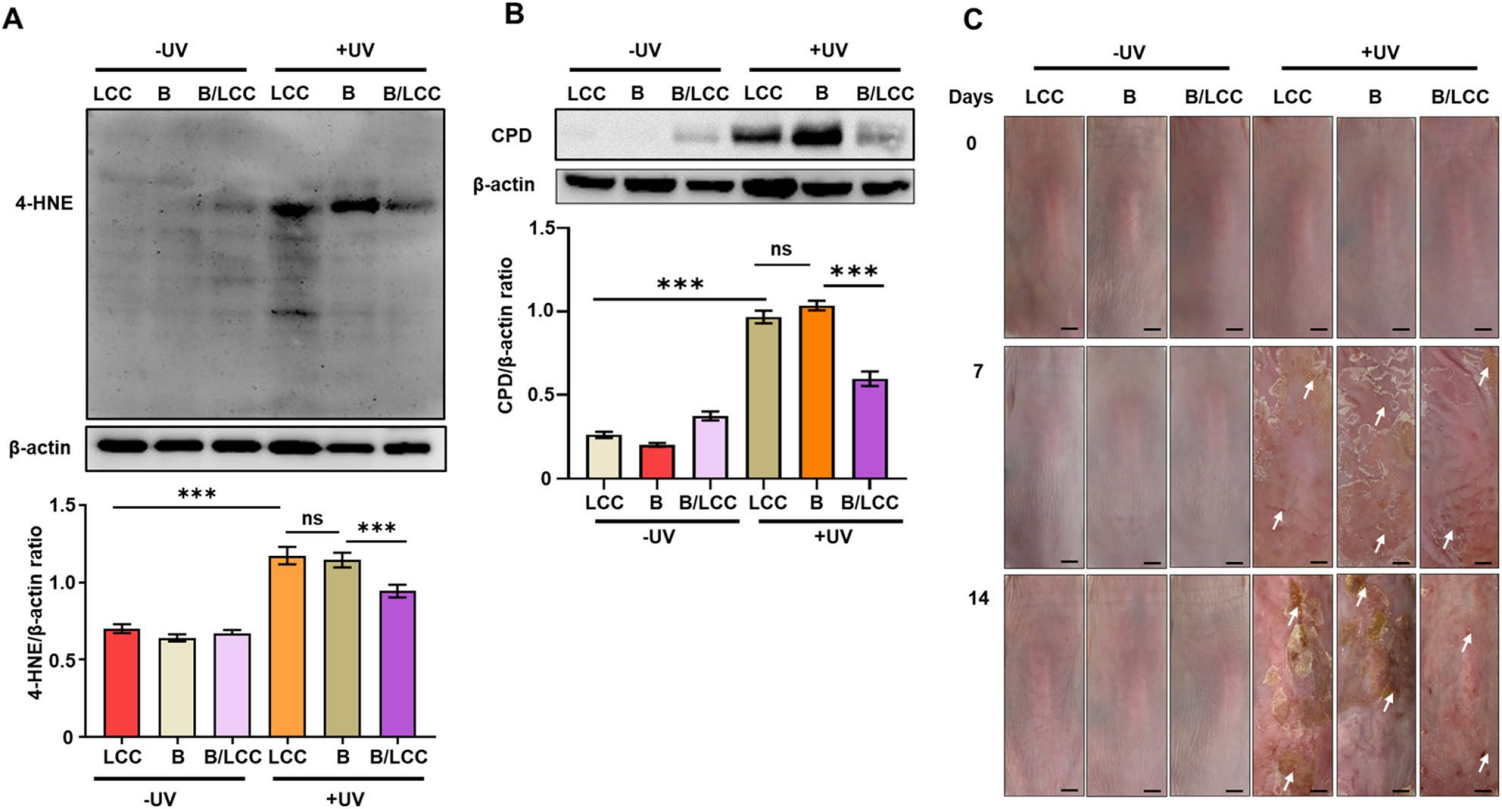
Effect of *S. epidermidis* in the presence of LCC on the UV-B-induced formation of 4-HNE, CPD and lesions. The dorsal skin of ICR mice topically applied with LCC alone, *S. epidermidis* ATCC 12228 alone (**B**), or *S. epidermidis* plus LCC (B/LCC) was irradiated with (+ UV) or without (− UV) UV-B. The images of protein bands of (**A**) 4-HNE or (**B**) CPD analyzed by western blot were displayed. The ratio of intensities of protein bands of 4-HNE or CPD normalized to β-actin was shown. (**C**) Morphologies of mouse skin irradiated with (+ UV) or without (− UV) 100 mJ/cm^2^ UV-B 0, 7, and 14 days after irradiation were shown. Skin lesions were indicated by white arrows. Data are the mean ± SD from three independent experiments. ***P < 0.001 (two-tailed t-test). ns = non-significant.

It has been documented that UV-B-induced free radicals can cause skin hyperplasia^31^, lipid peroxidation^32^ and the CPD formation^33^. We next assessed the influence of fermentation and electricity produced by *S. epidermidis* plus LCC on the UV-B-induced skin injuries. The recurrent exposure of UV-B significantly fostered the formation of 4-HNE and CPD on mouse skin topically applied with LCC or *S. epidermidis* alone. In addition, UV-B-induced epidermal hyperplasia, as characterized by an increase in epidermal thickness (Figure S1) and lesions (Fig. 3C), can be detected on mouse skin topically applied with LCC or *S. epidermidis* alone. However, the UV-B-induced the formation of 4-HNE, CPD, epidermal hyperplasia and lesions were considerably attenuated when mouse skin was topically applied with *S. epidermidis* plus LCC (Fig. 3, Figure S1). Topical application of PBS or LCC alone without *S. epidermidis* on mouse skin before UV-B irradiation exhibited the same levels of 4-HNE and CPD as well as skin lesions (Figure S2), indicating that LCC itself was insufficient to impede the UV-B-induced skin injuries. Data above defined a novel function for the repurposed LCC in conjunction with *S. epidermidis* for suppressing UV-B-induced skin injuries.

### The *S. epidermidis* S2 isolate with low expression of *pdh* and *pta* genes was poorly electrogenic

**Figure 4:**
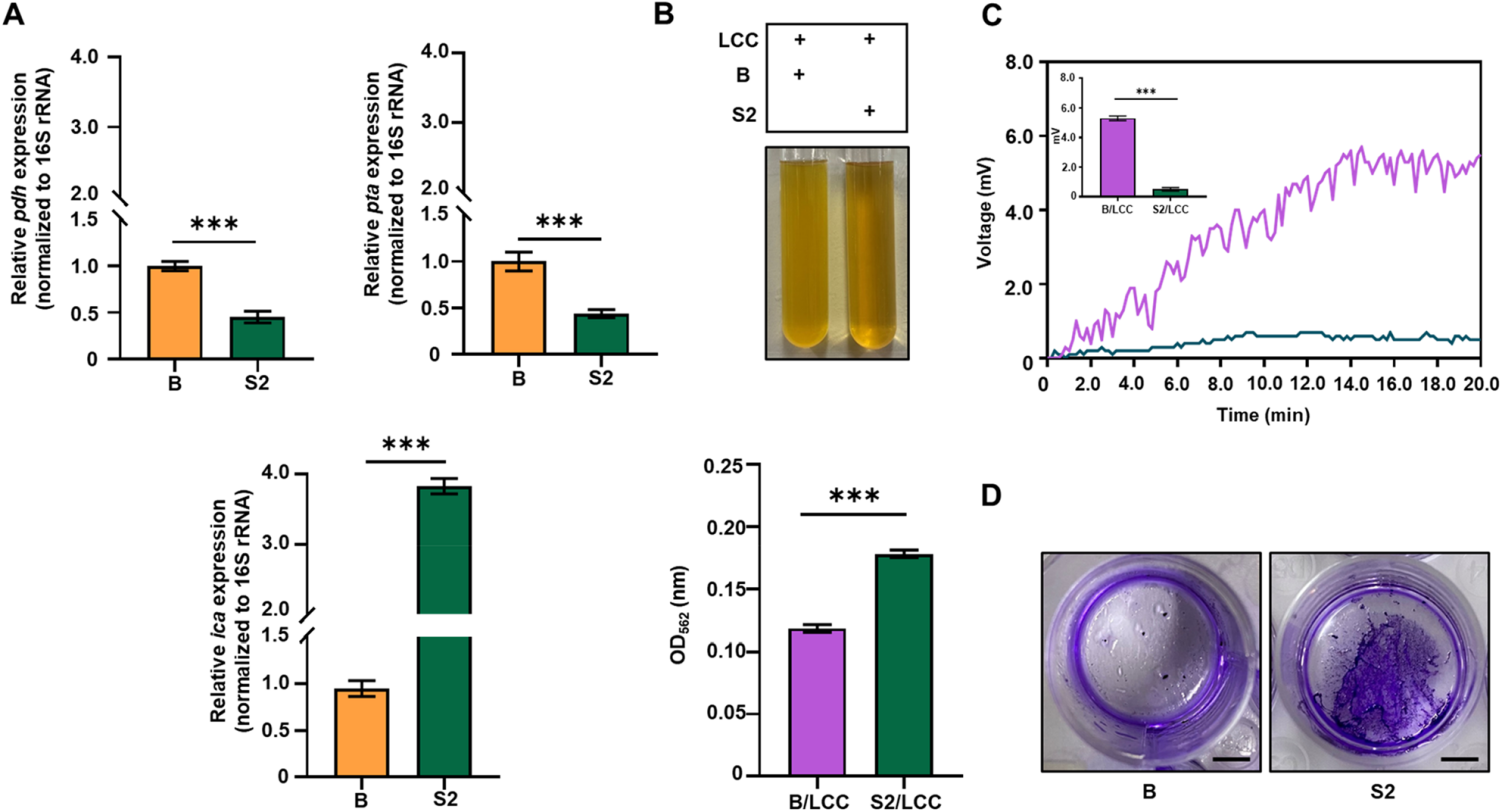
The gene (*pdh*, *pta*, and *icaA*) expression, electricity and biofilm formation in *S. epidermidis* ATCC 12228 and S2 isolate. (**A**) Relative expression of *pdh*, *pta*, and *icaA* genes normalized to 16S rRNA was analyzed by the RT-qPCR. (**B**) LCC fermentation for 12 h in rich media was quantified by measurement of OD_562_. (**C**) Electricity production of bacteria in the presence of 2% LCC was analyzed by voltage changes (mV) in an in vitro chamber. (**D**) Bacterial biofilms were stained by crystal violet after culture of bacteria in TSB on a 24-well plate for 48 h. B/LCC: *S. epidermidis* ATCC 12228 plus 2% LCC; S2/LCC: *S. epidermidis* S2 isolate plus 2% LCC. Scale bars = 1 cm. Data are the mean ± SD from three separate experiments. **P < 0.01; ***P < 0.001 (two-tailed t-test).

Since LCC effectively induced *S. epidermidis* to undergo fermentation and yield electricity, we next determined the expression of genes related to fermentation, acetate production, and biofilm formation in *S. epidermidis* ATCC 12228 and a *S. epidermidis* S2 strain isolated from human skin. The *pdh* gene encodes for pyruvate dehydrogenase, which catalyzes pyruvate to acetyl coenzyme A (acetyl-CoA) at the upstream site of the fermentation pathway^34^. The *pta* gene encoding for phosphate acetyltransferase is involved in the conversion of acetyl-CoA to acetate^35^. The *ica*A gene encoding for intracellular adhesion A has been well characterized in the engagement of the biofilm formation in *S. epidermidis*^36,37^. The 16 s rRNA sequence of *S. epidermidis* S2 isolate shared 99.8% identity to that of *S. epidermidis* ATCC 12228 (Table S1). However, the expressions of *pdh* and *pta* genes in *S. epidermidis* S2 isolate were much lower than those in *S. epidermidis* ATCC 12228 (Fig. 4A). Furthermore, the activity of LCC fermentation monitored by OD_562_ reduction of phenol red-containing rich media for *S. epidermidis* S2 isolate was relatively low compared to that in *S. epidermidis* ATCC 12228 (Fig. 4B). The high expression of *icaA* gene (Fig. 4A) and obvious biofilms (Fig. 4D) were detected in *S. epidermidis* S2 isolate, but not in the non-biofilm forming bacterial strain, *S. epidermidis* ATCC 12228. Interestingly, unlike *S. epidermidis* ATCC 12228, the *S. epidermidis* S2 isolate that expressed low levels of *pdh* and *pta* genes was poorly electrogenic. As shown in Fig. 4C, consistent with Fig. 2, a peak voltage of approximately 6 mV was detected in media with *S. epidermidis* ATCC 12228 plus LCC whereas little or no voltage change was measured in *S. epidermidis* S2 isolate plus LCC. Collectively, the *pdh* and *pta* genes may participate in fermentation and electricity production of *S. epidermidis* triggered by LCC.

### LCC plus *S. epidermidis* S2 isolate did not confer protection against UV-B-induced skin injuries

**Figure 5:**
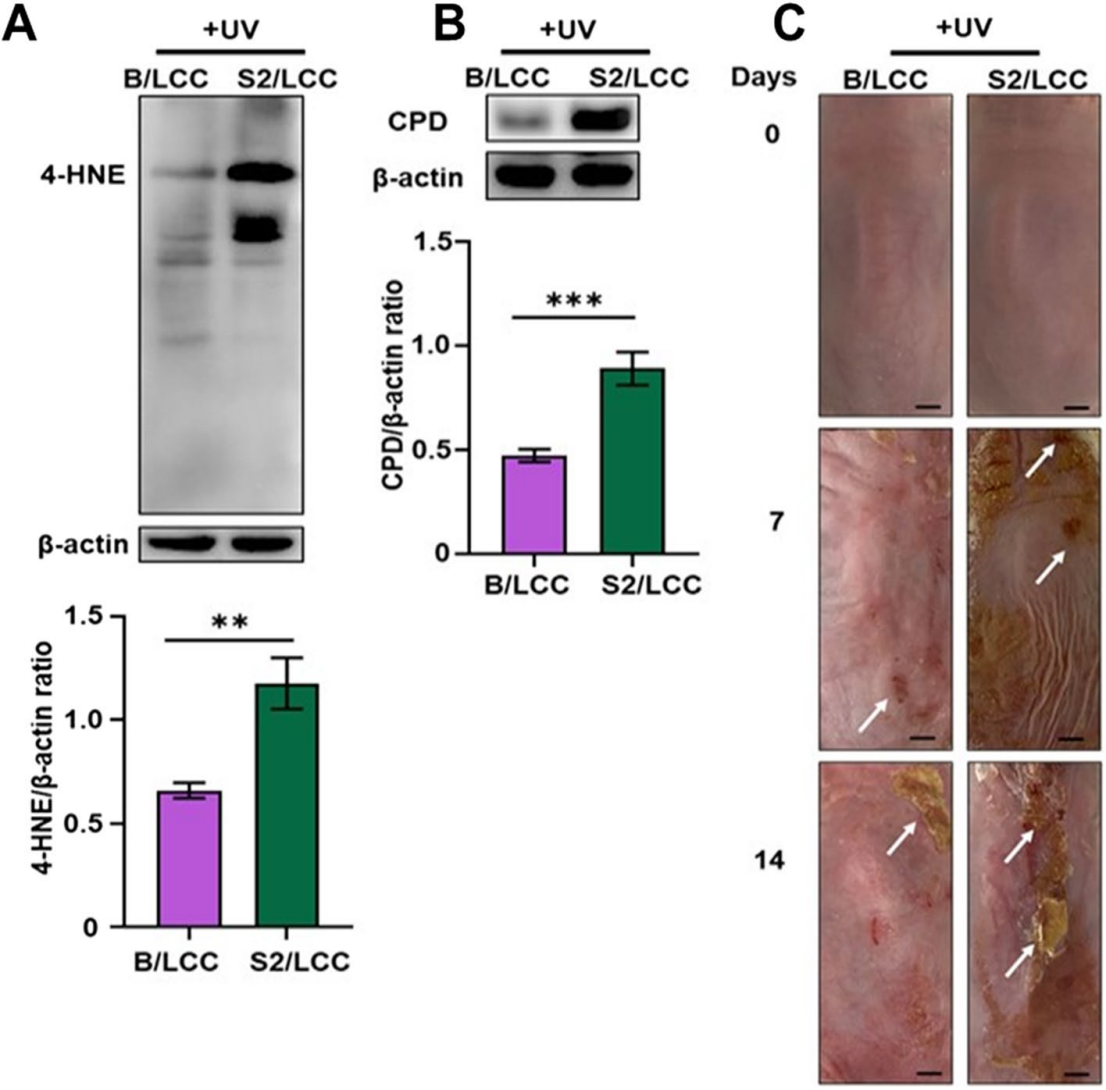
The UV-B-induced formation of 4-HNE, CPD and lesions in mouse skin applied with LCC in combination with *S. epidermidis* S2 isolate. The dorsal skin of ICR mice was topically applied with *S. epidermidis* ATCC 12228 (B/LCC) or *S. epidermidis* S2 (S2/LCC) in the presence of 2% LCC. The levels of (**A**) 4-HNE, (**B**) CPD related to β-actin in western blot analysis and (**C**) lesions (at 0, 7, and 14 days post-irradiation) on mouse skin irradiated with 100 mJ/cm^2^ UV-B (+ UV) were shown. Skin lesions were indicated by white arrows. Data are the mean ± SD from three separate experiments. **P < 0.01; ***P < 0.001 (two-tailed t-test).

To confirm the critical roles of *pdh* and *pta* genes in LCC fermentation of *S. epidermidis* against UV-B in vivo, dorsal skin of ICR mice was topically applied with *S. epidermidis* ATCC 12228 or S2 isolate in the presence of LCC before UV-B irradiation. The levels of 4-HNE and CPD in mouse skin were measured by western blot. In agreement with data in Fig. 3, after UV-B irradiation, the formation of 4-HNE, CPD and skin lesions were detected at low levels and moderate on mouse skin topically applied with *S. epidermidis* plus LCC. However, when mouse skin was topically applied with *S. epidermidis* S2 isolate plus LCC (Fig. 5A–C), UV-B induced significantly increasing levels of 4-HNE and CPD as well as skin lesions. Since the expressions of *pdh* and *pta* genes in *S. epidermidis* S2 isolate were much lower than those in *S. epidermidis* ATCC 12228 (Fig. 4), the expressions of *pdh* and *pta* genes may mediate the LCC-triggered promotion of *S. epidermidis* against skin injuries caused by UV-B.

## Discussion

LCC is an ester obtained from the reaction of the coconut alcohol-derived fatty acids with a mixture of caprylic acid and capric acid^38^. Both caprylic acid and capric acid, as medium-chain fatty acids, can be extracted from coconut oil and have been used as ingredients for skincare formulation to protect against UV radiation^39,40^. It has been reported that addition of caprylic acid into the fermentation process enhanced acetate production^41^. Coconut oil has been used as a fuel for electricity production^42^. Although many medium-chain fatty acids exhibited potent bactericidal activities^43^, our result in Figure S3 demonstrated that LCC did not change the growth of *S. epidermidis*. Compared to other INCI-registered compounds (ININ, PDS and PETIS), LCC displayed higher activity in promoting fermentation and electricity production of *S. epidermidis* (Fig. 1). Biofilms are electroactive and can promote the electricity production of bacteria^44^. By using *S. epidermidis* ATCC 12228, a non-biofilm forming strain, we demonstrated that skin bacteria can produce electricity without biofilm formation on electrodes. Data in our recent publication has revealed that addition of glycerol into the *S. epidermidis* culture can induce fermentation and instantly produce detectable electricity, highlighting a possible mechanism that skin bacteria underwent fermentation to accumulate SCFAs as electron donors to intensify electricity^18^.

Results in Fig. 1 demonstrated that the electricity measured by changes in voltage and currents was considerably produced when the anode was pipetted with *S. epidermidis* ATCC 12228 plus 2% LCC. However, the electricity produced by bacteria plus LCC was largely reduced in the *S. epidermidis* S2 isolate which expressed lower levels of *pdh* and *pta* genes (Fig. 3). The data indicated that proteins corresponding to *pdh* and *pta* genes may play a role in electricity production of *S. epidermidis* in the presence of LCC. Electrons are derived by the reduction reaction of NAD^+^ to NADH in the metabolic pathway of bacterial fermentation^45^. The conversion of NAD^+^ to NADH is involved in pyruvate dehydrogenase (*pdh*), which initiates the process of electron transport chain^45,46^. Previous studies have shown that electricity production of *Shewanella oneidensis* MR-1, a representative electrochemically active bacterium (EAB) extensively studied in the laboratory, was mediated by activation of NAD^+^-linked PDH^47^. Phosphate acetyltransferase (*pta*) can catalyze the conversion of acetyl-CoA to acetate, a known electron donor. A *pta* knockout strain of *Shewanella oneidensis* strain has been used to study the effect of electricity on bacterial fermentation^48^. The protective effect of bacterial fermentation on the suppression of the UV-B-induced formation of 4-HNE and CPD was remarkably diminished when mouse skin was topically applied with LCC and *S. epidermidis* S2 isolate which yielded low electricity and expressed lower levels of *pdh* and *pta* genes (Fig. 5). The data suggested that SCFAs and electricity induced by LCC fermentation of *S. epidermidis* may synergistically provide mice protection from UV-B injuries. Future studies will include the construction of a *pdh* knockout *S. epidermidis* strain and investigation of the essential role of *pdh* gene in *S. epidermidis* for production of electricity against UV-B injuries.

4-HNE is known to be genotoxic and damages DNA by producing bulky 1,*N*^2^-propano-2′-deoxyguanosine adducts^49^. Here, we showed that 4-HNE and CPD were formed when mouse skin was constantly bombarded with UV-B. Results in our previous studies have demonstrated that butyrate produced by glycerol fermentation of *S. epidermidis* can down-regulate UV-B-induced pro-inflammatory interleukin (IL)-6 cytokines through SCFA receptor 2 (FFAR2)^50^. Thus, skin bacteria may take advantage of endogenous glycerol as a carbon source to provoke fermentation and simultaneously produced SCFAs and electrons. Besides being electron donors, SCFAs may regulate FFAR2 and/or histone deacetylases (HDAC)^51^ to mitigate the inflammation induced by UV irradiation. Electrons may play a key role in neutralizing free radicals generated by UV irradiation. It has been shown that UV light can mediate nitrate, a constituent of sweat in skin, to generate free radicals^52^, which can subsequently induce lipid peroxidation to produce 4-HNE^53^. However, commensal bacteria can utilize nitrate as an electron acceptor for energy transduction^54^. For example, under anoxic or oxygen-depleted conduction, *Pseudomonas* species can mediate denitrification by using nitrate as an electron acceptor to convert nitrate to nitrogenous gases^55^. Thus, a possible mechanism behind the protective effect of *S. epidermidis* plus LCC on UV-B skin injuries is that LCC triggers bacteria to produce electrons, activating bacterial denitrification to reduce UV-B-induced free radicals.

In summary, prebiotics for skin probiotic bacteria have been not defined by the Food and Drug Administration (FAD) even though they may become novel therapeutics for treatments of skin disorders via induction of fermentation of skin bacteria. Our study here demonstrated an approach by repurposing INCI-registered compounds as skin prebiotics and revealed their capabilities of generating electricity from skin bacteria to combat UV-B-induced skin damages.

## Supplementary information

Supplementary Information.
