# Supplementary material for: Repurposing INCI-registered compounds as skin prebiotics for probiotic Staphylococcus epidermidis against UV-B
Source: Sci Rep. 2020 Dec 9;10:21585. doi: 10.1038/s41598-020-78132-5 (PMC7725810; doi:10.1038/s41598-020-78132-5)
Supplement: Supplementary file 1 — Supplementary Information. [file 41598_2020_78132_MOESM1_ESM.docx]

**Supplemental Information**

**Repurposing INCI-registered compounds as skin prebiotics for probiotic *Staphylococcus epidermidis* against UV-B**

Arun Balasubramaniam^1^, Prakoso Adi^1^, Do Thi Tra My^1^, Sunita Keshari^2^_,_ Raman Sankar^3^, Chien-Lung Chen^4^, Chun-Ming Huang^1*^

^1^Department of Biomedical Sciences and Engineering, National Central University, Taoyuan, Taiwan.

^2^Department of Life Sciences, National Central University, Taoyuan, Taiwan.

^3^Institute of Physics, Academia Sinica, Nankang, Taipei, Taiwan.

^4^Division of Nephrology, Landseed International Hospital, Taoyuan, Taiwan.

Running title: Repurposing skin prebiotics for probiotic *S. epidermidis* against UV-B

^*^Corresponding author. E-mail address: [chunming@ncu.edu.tw](mailto:chunming@ncu.edu.tw) (C.M.H.)

Tel.: +886-3-422-7151 (ext. 36104);

Fax: +886-3-425-3427

**Supplementary materials and methods**

**Hematoxylin and Eosin (H&E) staining:** The histological review was done to evaluate the thickness of the epidermis. After 2 weeks of UV-B treatment, dorsal skin samples from the experimental groups were collected, fixed with 10% formalin, and encased in paraffin. The epidermal region of the skin samples has been stained with H&E to define the thickness of the region. The epidermal thickness was calculated at 10x magnification using the Olympus BX63 microscope (Olympus, Tokyo, Japan).

## Minimum bactericidal concentration (MBC)

2% LCC or 1x PBS was incubated with 10^7^ CFU/ml *S. epidermidis* ATCC 12228 in a 1.5 ml Eppendorf tube for 12 h at 37°C. Bacteria incubated with 2% LCC or 1x PBS was serially diluted 1: 10^0^-1:10^5^ in a 96 well plate after incubation. 10 μl of serially diluted bacteria were dropped on the surface of the TSB agar plate. The counting CFUs of bacteria was used for determining the number of bacteria.

**Table S1.** 16s rRNA sequence of *S. epidermidis* S2 isolate.

| **Colony** | **Nucleotide Sequence** | **Species** | **% Identity** |
| --- | --- | --- | --- |
| **S2** | GGACTGCGCTGCTATACATGCAGTCGAGCGAACAGACGAGGAGCTTGCTCCTCTGACGTTAGCGGCGGACGGGTGAGTAACACGTGGATAACCTACCTATAAGACTGGGATAACTTCGGGAAACCGGAGCTAATACCGGATAATATATTGAACCGCATGGTTCAATAGTGAAAGACGGTTTTGCTGTCACTTATAGATGGATCCGCGCCGCATTAGCTAGTTGGTAAGGTAACGGCTTACCAAGGCAACGATGCGTAGCCGACCTGAGAGGGTGATCGGCCACACTGGAACTGAGACACGGTCCAGACTCCTACGGGAGGCAGCAGTAGGGAATCTTCCGCAATGGGCGAAAGCCTGACGGAGCAACGCCGCGTGAGTGATGAAGGTCTTCGGATCGTAAAACTCTGTTATTAGGGAAGAACAAATGTGTAAGTAACTATGCACGTCTTGACGGTACCTAATCAGAAAGCCACGGCTAACTACGTGCCAGCAGCGGCCCGGTAATTAATA | *S. epidermidis* | 99.80 |

**Table S2.** Primers used for RT-qPCR.

| **Gene** | **Primer Sequence** |
| --- | --- |
| ***pdh*** | Forward 5’ CTTCTACTGATGTCGTTAATGCTTCTG 3’  Reverse 5’ GCAATTGCTTTGCGCATTG 3’ |
| ***pta*** | Forward 5’ GCTGAAGCAGATCAATTAGATCATG 3’  Reverse 5’ TTGCATCACCTTGAATTTTTGC 3’ |
| ***icaA*** | Forward 5’ GAACGCGCACTTGCTTACG 3’  Reverse 5’ AGCGTTTCAAATGCATCATTACTTA 3’ |
| ***16s*** | Forward 5’ ATGCACGTCTTGACGGTACCT 3’  Reverse 5’ TCCATGGCAGTTCTGCACGTA 3’ |


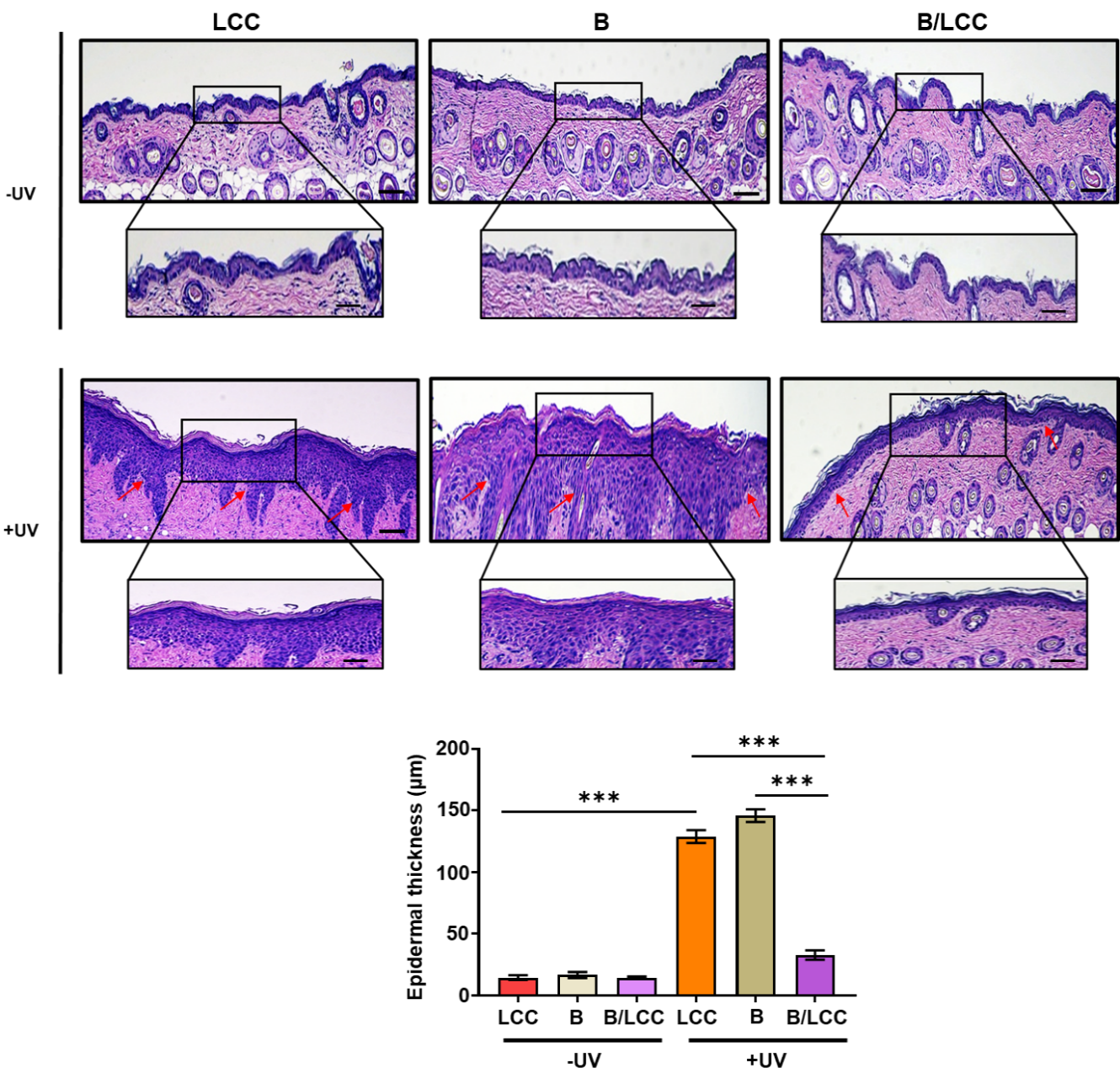


**Figure S1.** Histological analysis of non-irradiated and UV-B irradiated mouse skin. H&E-stained histological images of non-irradiated (-UV) and UV-B (+UV) irradiated mouse skin were topically applied with *S. epidermidis* ATCC 12228 (10^7^CFU/ml; B), 2% LCC, or *S. epidermidis* ATCC 12228 plus LCC (B/LCC). The epidermal thickness in zoomed regions was quantified and presented as mean ± SD. Scale bars = 100 µm. Representative images in one of three independent experiments are shown. ****P* < 0.001 (two-tailed t-test).


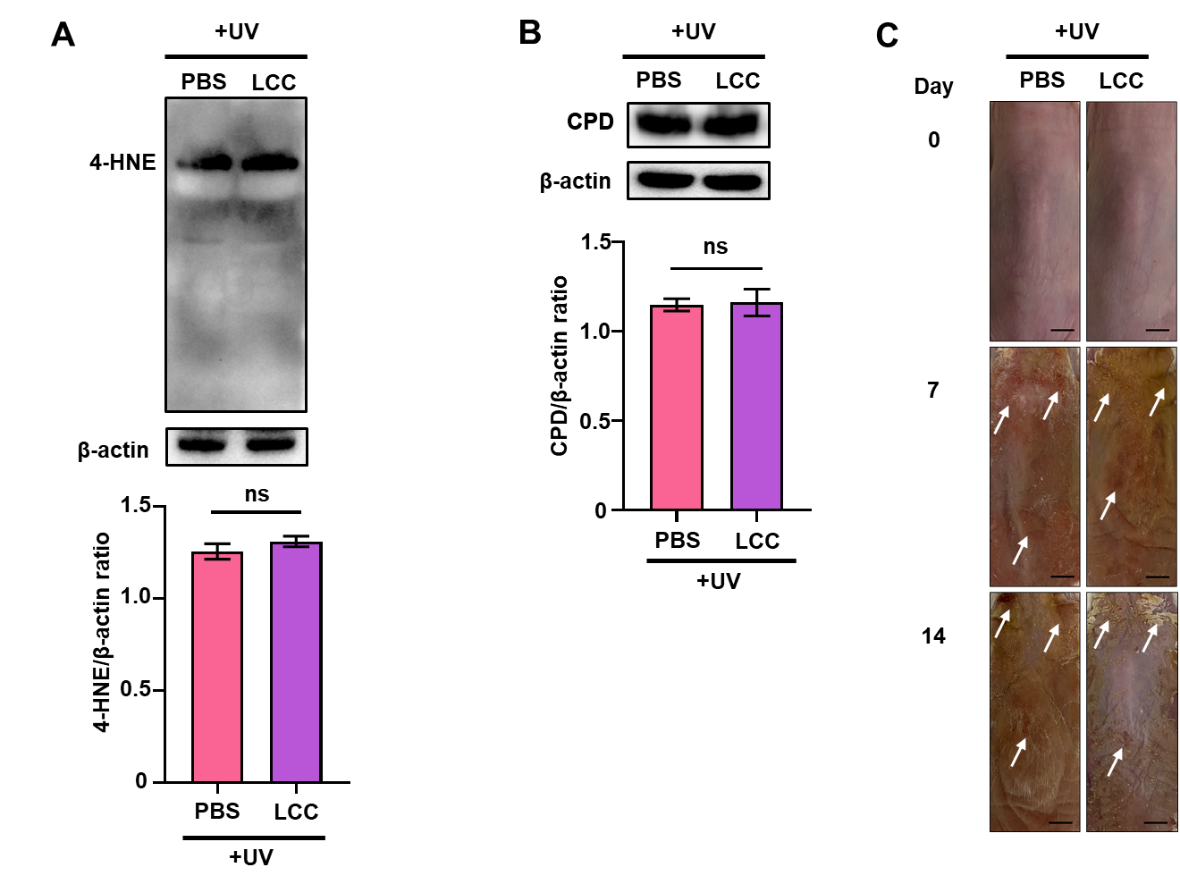


**Figure S2.** No effect of LCC on the UV-B-induced formation of 4-HNE and CPD. The level of 4-HNE (A) or CPD (B) in UV-B (+UV) irradiated mouse skin topically applies with PBS or 2% LCC was detected by western blot. The intensities of protein bands normalized to β-actin were quantified (mean ± SD; in triplicate) using ImageJ. ns = non-significant. Representative images of mouse skin were captured on day 0, 7, and 14. Skin lesions were indicated by white arrows.

**
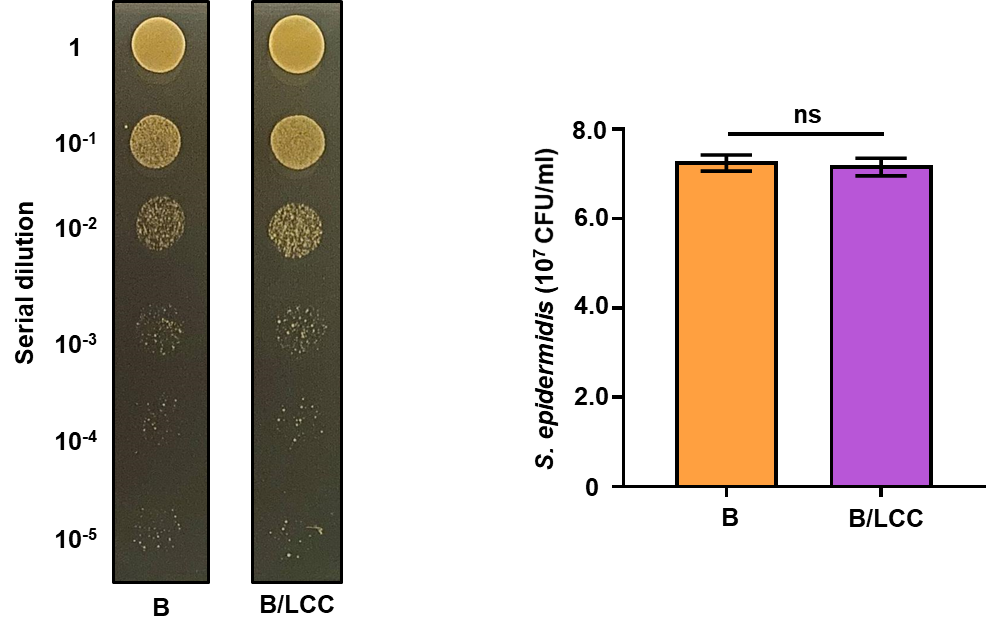
**

**Figure S3.** No effect of LCC on the growth of *S. epidermidis*. *S. epidermidis* ATCC 12228 (10^7^ CFU/ml) was cultured with and without 2% LCC for 12 h. Serially diluted (1:10^0^-1:10^5^) bacterial culture was placed on TSB agar plates for CFU counts. Data are the mean ± SD of experiments performed in triplicate. ****P* < 0.001 (two-tailed t-test). ns = non-significant.


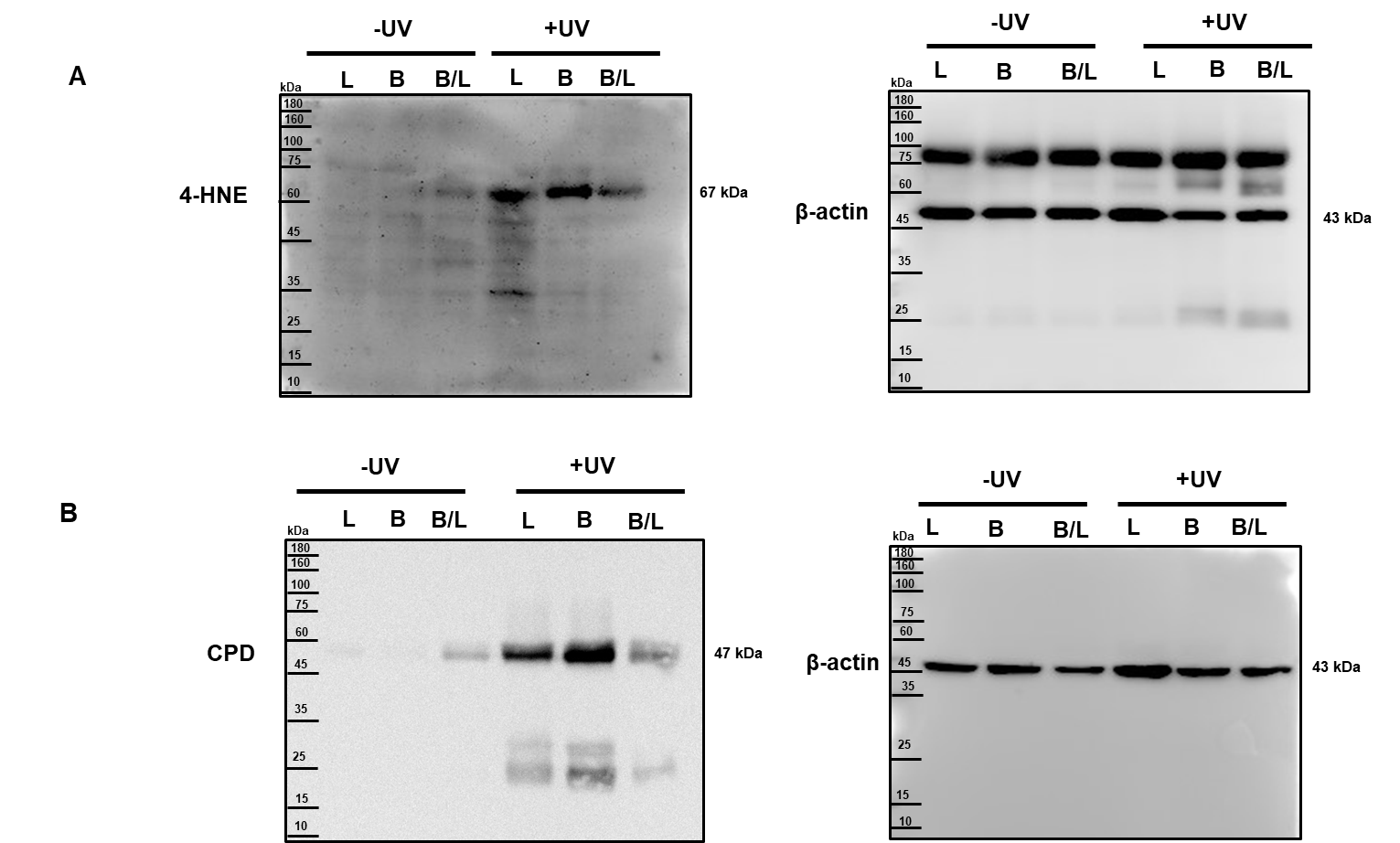


**Figure S4.** The uncropped western blot images of Figure 3A, and B. The lanes from top to bottom of each blot represent the size of protein ladder (kDa); The protein band levels of 4-HNE & β-actin (A) and CPD & β-actin (B) in the skin tissue of mice treated with and without UV.


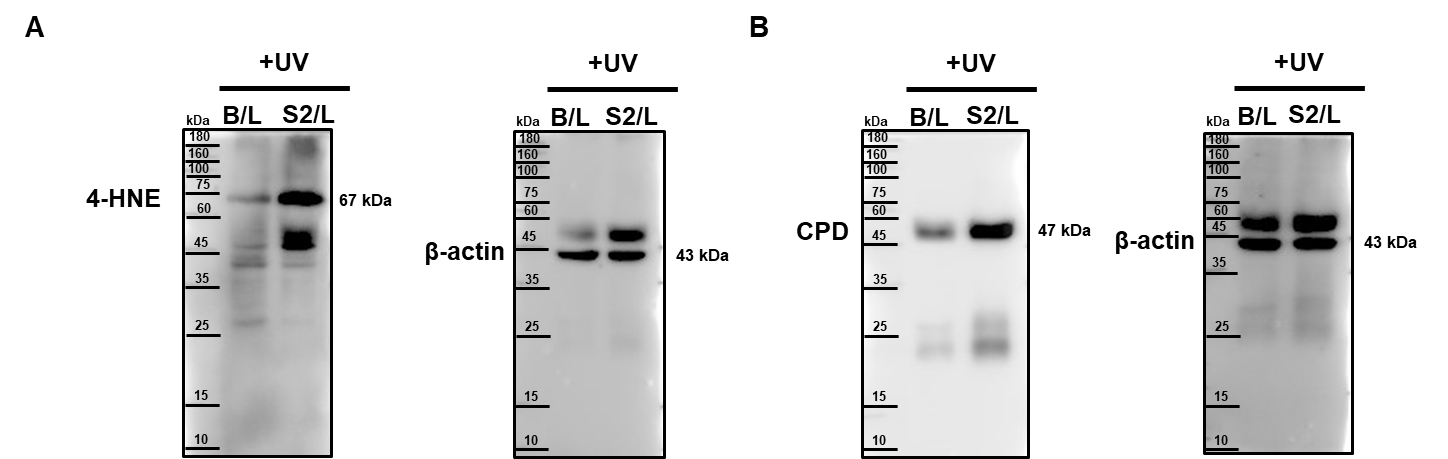


**Figure S5.** The uncropped western blot images of Figure 5A, and B. The lanes from top to bottom of each blot represent the size of protein ladder (kDa); The protein band levels of 4-HNE & β-actin (A) and CPD & β-actin (B) in the skin tissue of mice treated with UV.


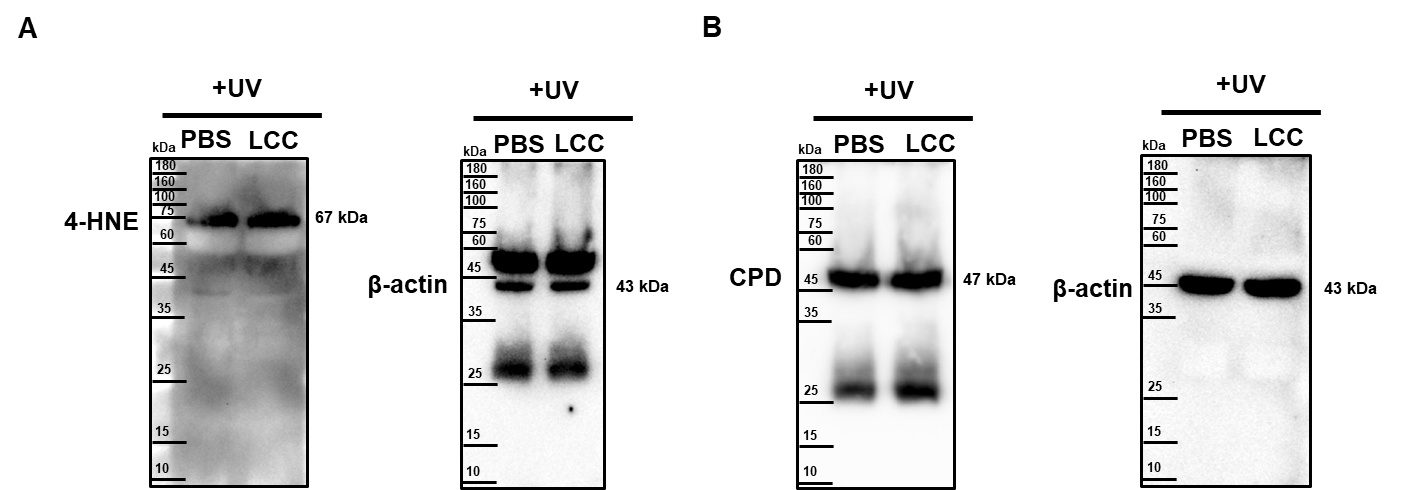


**Figure S6.** The uncropped western blot images of Figure S2A, and B. The lanes from top to bottom of each blot represent the size of protein ladder (kDa); The protein band levels of 4-HNE & β-actin (A) and CPD & β-actin (B) in the skin tissue of mice treated with UV.
